# Doctors and Their Work

**Published:** 1904-09

**Authors:** 


**Doctors and their Work.** By ROBERT BRUDENELL CARTER. Pp. vi., 316. London: Smith, Elder & Co. 1903.

This book deals with many of the problems which arise in the intercourse between doctors and their patients; and contains the author's views on the professional education of students, the aims of medicine, the daily life of medical practitioners, specialism, lady doctors, professional etiquette, and other cognate subjects. All of these are treated with thoughtfulness and judgment; the author's experience is on almost every page, and as Mr. Carter has been in our ranks for more than half a century, and has, during that time, had excellent opportunities of forming opinions on these matters, we may consider his advice and conclusions well worth having. There is very little in the book that any of us would disagree with, for on any question about which men are likely to differ he has a judicial and open mind.

His style is clear and good: occasionally one could wish the sentences were shorter, but they are never involved. Here and there we find criticisms and statements with which we cannot agree, as, for example, on p. 31, when he sharply condemns a schoolmaster for using the expression "the higher we advance." "He did not know," writes Mr. Carter, "that, although we may *rise* 'higher,' we can only *advance* 'farther.'" But the derivation of the word advance would justify the schoolmaster, and indeed Milton and many other masters of English have used it in this way. We might venture to criticise such sentences as "functions to which various organs are subservient," and we do not think Demetrius the silversmith at Ephesus a good example of an "anti," to be classed with anti-vaccinationists and anti-vivisectionists.

But apart from some unimportant points, we can clearly recommend this book as a just and thoughtful expression of opinion from a man who has gained great experience in the work and duties of our profession.

